# Chemokine receptors in vascular biology: a review of current evidence, implications, and therapeutic targets for hypertension

**DOI:** 10.1042/CS20257214

**Published:** 2025-08-26

**Authors:** Luis Henrique Oliveira de Moraes, Tyler Beling, Gustavo Felix Pimenta, Thiago Bruder-Nascimento

**Affiliations:** 1 Physiological Sciences, Universidade Federal de São Carlos, São Carlos, São Paulo, Brazil; 2 Physiology and Cell Biology, Frederick P. Whiddon College of Medicine, University of South Alabama, Mobile, AL, U.S.A; 3 Department of Pharmacology, University of Sao Paulo, Sao Paulo, Brazil

**Keywords:** chemokines, hypertension, vascular biology

## Abstract

Cardiovascular diseases (CVDs) remain the leading cause of death worldwide, including in the United States. Risk factors such as high cholesterol, diabetes, obesity, smoking, physical inactivity, and hypertension contribute significantly to their development. Emerging evidence highlights a central role for chemokines—small signaling molecules that guide immune cell migration to sites of infection, inflammation, or tissue damage—in the initiation and progression of hypertension. This positions chemokines and their receptors as promising pharmacological targets for blood pressure regulation and vascular protection. In this review, we explore the therapeutic potential of targeting chemokines and their receptors and summarize the main strategies reported in the literature for managing hypertension through these pathways. For this purpose, an analysis of drugs that act on the most relevant receptors at the preclinical and clinical levels was performed. After this analysis, their mechanisms of action, selectivity, and possible adverse effects were discussed. In conclusion, we reinforce that the modulation of chemokines and their receptors represents a promising approach in the control of CVDs, especially hypertension, although further clinical studies are needed to validate the efficacy and safety of this strategy, considering possible impacts on other essential immune responses.

## Introduction

According to the American Heart Association’s 2024 report, approximately 127.9 million adults in the United States (48.6%) are affected by cardiovascular diseases (CVDs), including conditions such as coronary heart disease, heart failure, and hypertension [1].

Furthermore, reports from the World Health Organization in September 2023 indicate that one in three people in the world suffers from hypertension [2,3]. CVDs remain the leading global cause of death, accounting for approximately 32% of all deaths in the world, which represents approximately 19.8 million deaths [3].

Although significant progress has been made in understanding the mechanisms underlying the development of CVDs, many critical gaps remain. One emerging area of interest is the role of the immune system in regulating cardiovascular function. Increasing evidence suggests that an exaggerated or dysregulated immune response significantly contributes to the pathogenesis of CVDs. In particular, the overproduction of pro-inflammatory cytokines and chemokines—such as interleukin-6 (IL-6) [4], tumor necrosis factor-alpha (TNF-α) [5], C-C motif chemokine ligand 2 (CCL2) also known as monocyte chemoattractant protein-1 (MCP-1) [6], and CCL5 [7]—can trigger and sustain chronic inflammation in key organs, including the kidneys, heart, and vasculature. This chronic inflammatory state disrupts vascular homeostasis, promotes endothelial dysfunction, and contributes to renal and cardiac injury, all of which are closely linked to hypertension. Hypertension, in turn, plays a central role in both the initiation and progression of CVDs [8]. Furthermore, both preclinical [7,9–11] and clinical studies [12–14] have explored the potential of targeting chemokine pathways in the treatment of atherosclerosis, hypertension, and other cardiovascular comorbidities. Among these, chemokines and their receptors—particularly CCR2, CXCR2, and CCR5—have emerged as promising therapeutic targets for CVD.

Thus, this review article seeks to explore the role of chemokines and their receptors in the genesis and progression of hypertension, elucidating the direct mechanisms of chemokines inducing vascular injury and describing their participation in recruiting immune cells into the inflamed or injured tissues, especially the vasculature. It further critically analyzes chemokines and their receptors as potential therapeutic targets, focusing on emerging inhibitors and novel approaches for the management of hypertension.

## Pioneering insights: chemokines in the onset of CVDs

One of the first studies exploring the relationship between chemokines and CVDs appeared in the late 1990s and early 2000s. These studies investigated the role of chemokines in vascular inflammation and their respective influence on the development of cerebrovascular diseases [15,16], hypertension [16], and atherosclerosis [17].

An example of the first studies is the article by Wang and collaborators, published in 1995, which investigated the expression of the messenger RNA of CCL2 in the ischemic cortex of rats after occlusion of the middle cerebral artery. This pioneering study highlighted the role of CCL2 as a local mediator of monocyte attraction, providing foundational insights into the inflammatory processes that drive vascular damage in conditions like ischemic infarction.

CCL2 is a chemokine of the CC subfamily that plays a crucial role in the immune system and inflammatory processes. This molecule is responsible for attracting monocytes, macrophages, and, to a lesser extent, T lymphocytes to sites of inflammation or tissue injury [18].

It is also seen that chemokines play an essential role in the pathophysiology of hypertension, mediating vascular inflammation and dysfunction [19]. When hemodynamic stress occurs, activated endothelial cells begin to express chemokines such as CCL2 [20] and CXCL8 [21], attracting monocytes and neutrophils to the blood vessels. These monocytes differentiate into macrophages, secreting inflammatory cytokines that exacerbate inflammation. This process contributes to vascular remodeling, resulting in arterial stiffness and increased vascular resistance, characteristics of hypertension [22]. In addition, chemokines activate the renin–angiotensin–aldosterone system (RAAS), promoting inflammation and renal fibrosis, which worsens sodium retention and increases blood pressure (BP) [23].

The recruitment of immune cells to organs such as the heart and kidneys also causes fibrosis, worsening the function of these organs [24]. Although the role of chemokines in CVD— particularly ischemic conditions—has been extensively studied, their involvement in hypertension remains relatively underexplored. In the sections below, we will examine the potential mechanisms by which chemokines and their receptors contribute to the development of hypertension. Special attention will be given to CCR2, CCR5, CXCR1, CXCR2, and CXCR4, which are among the most thoroughly characterized to date.

## Chemokines and hypertension

**Figure 1:**
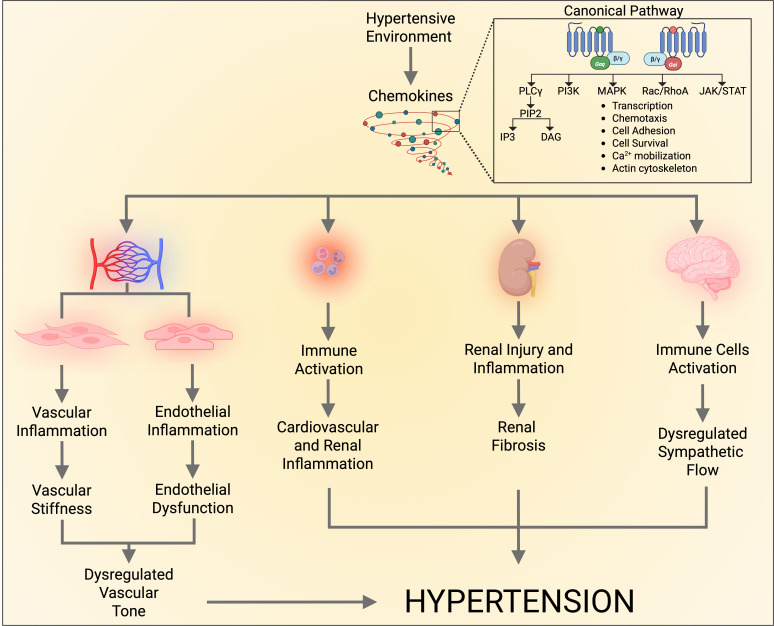
Hypertension is associated with elevated chemokine levels. Chemokines signal through G protein–coupled receptors (Gαi or Gαq), which are expressed in key organs involved in blood pressure regulation. Through this canonical pathway, chemokines exert direct effects on the vasculature, kidneys, and brain. In addition to driving immune activation, these effects contribute to cardiovascular dysfunction and hypertension. DAG, diacylglycerol; IP3, inositol trisphosphate; JAK, Janus kinase; MAPK, mitogen-activated protein kinase; PI3K, phosphoinositide 3-kinase; PIP2, phosphatidylinositol 4,5-bisphosphate; PLCγ, phospholipase C γ; Rac, ras-related C3 botulinum toxin substrate GTPase; RhoA, ras homolog family member A; STAT, signal transducer and activator of transcription.

Research from our group and others has demonstrated that chemokines can bind to their receptors within the vasculature, initiating vascular dysfunction, inflammation, and structural remodeling [25]. These receptors are G protein–coupled receptors, primarily associated with the Gi and Gq protein families. Upon activation, they initiate specific intracellular signaling cascades. Chemokines and their receptors are classified based on the conserved cysteine residue patterns within their structure [26] (Table 1 and Figure 1).

**Table 1:** Classification of chemokines according to the cysteine position

Group of chemokines	Cysteine position	Examples
CC chemokines	Adjacent cysteines	CCL2 (MCP-1), CCL4, and CCL5 (RANTES)
CXC chemokines	Cysteines separated by one amino acid	CXCL8 (IL-8) and CXCL10 (IP-10)
CX3C chemokines	Two cysteines separated by three amino acids (C–X–X–X–C motif)	CX3CL1 (fractalkine)
XC chemokines	Lacking one of the first two cysteines	XCL1 (lymphotactin)

Although chemokines are expressed in various cell types and organs, their expression levels differ significantly across tissues. For instance, vascular smooth muscle cells (VSMCs) express high levels of CCR5, CCR8, and CX3CR1 [27–29], particularly under inflammatory conditions. In contrast, endothelial cells largely express several other chemokine receptors including CCR4, CCR5, CCR6, CXCR2, CXCR4, and CXCR5 [30], and CXCR4, which plays a key role in pulmonary arterial hypertension (PAH) through an autocrine signaling mechanism [31]. These observations suggest that the function of chemokine receptors is highly cell type–specific, with distinct roles depending on the cellular context.

This inflammatory process contributes to endothelial dysfunction, increasing vascular tone and promoting remodeling [7,32,33]. Notably, this amplified vasoconstrictor response can occur even in the absence of direct immune cell infiltration into the vascular wall, suggesting that inflammatory mediators alone may be sufficient to drive these changes. For instance, various nonleukocytic cell types express chemokine receptors, such as neurons, astrocytes, epithelial cells, endothelial cells, mesenchymal cells, VSMCs, and cancer cells. This explains the contribution of chemokines to cell survival, proliferation, differentiation, tumor growth as well as metastasis [19,33,34]. For example, CXCR5 and CCR7 play a crucial role in development, as they regulate the homing of lymphoid tissue inducer cells, while other chemokine interactions (CXCL12/SDF-1 and CXCR4) directly affect angiogenic activity either positively or negatively, by regulating endothelial cells during angiogenesis [35,36]. Interestingly, chemokines can also contribute to vasodilation/vasoconstriction by triggering Ca^2+^ influx. Chemokine-stimulated cytosolic Ca^2+^ spikes are caused by CCR5 and CCR1/CCR2, on VSMCs. These chemokine-mediated signals can furthermore sustain intracellular Ca^2+^ in VSMCs, via receptor-operated, non-voltage gated channels, ultimately leading to vasoconstriction [37–39]. Thus, the role of chemokines in hypertension has garnered significant attention due to their dual capacity to modulate immune cell function and directly influence key organs involved in BP regulation.

In addition to their role in vascular damage and dysfunction, chemokines are key modulators of organs and systems involved in BP regulation. Notably, they influence renal function by promoting inflammatory and fibrotic responses in kidney cells [40,41] and also affect central nervous system (CNS) activity [19].

In the kidneys of rodents treated with angiotensin II (Ang II), chemokines such as CCL2 and CXCL10 are up-regulated. These chemokines facilitate the recruitment of pro-inflammatory immune cells to vascular and renal tissues, resulting in inflammation [41]. This inflammatory response promotes renal fibrosis, increases oxidative stress, and impairs sodium excretion, all of which contribute to the development and progression of hypertension [40,42].

Similarly, in the CNS, chemokines such as CCL5 and CXCL1 are implicated in neuroinflammation associated with hypertension. These chemokines promote immune cell infiltration into brain regions that are critical for BP regulation, influencing sympathetic nervous system activity and further contributing to hypertensive pathology [19]. An overview of these mechanisms is illustrated in Figure 1.

## Chemokine receptors as therapeutic targets

In this section, we will discuss the main chemokine receptors involved in the development of hypertension. Understanding these receptors and their interactions with specific ligands is fundamental to create new therapeutic approaches against hypertension. The literature mainly highlights the receptors CCR2, CCR5, CXCR1, CXCR2, and CXCR4 as the most relevant for the progression of hypertension.

### CCR2

The CCL2-CCR2 axis is one of the major chemokines signaling pathways that has received special attention because of its function in the development and progression of CVDs. Evidence shows that elevated levels of circulating CCL2 are associated with increased long-term cardiovascular mortality, even in individuals without significant CVDs [43]. Clinical studies with colchicine, a blocker of the CCL2-CCR2 axis [44], have demonstrated the efficacy of anti-inflammatory treatment in preventing CVDs, reinforcing the need for further research on the role of the chemokine CCL2 and its receptor, CCR2, in conditions such as hypertension [45].

As an example, Tucker et al. [14] studied the effects of colchicine on transcoronary chemokine (TC) levels in patients with acute coronary syndrome (ACS). In this study, there were three experimental groups, of which 12 patients with ACS and 13 with stable angina pectoris (SAP) received treatment with colchicine, while the third group with 13 more participants with ACS did not receive treatment. Among the results, it was seen that the plasma levels of CCL2 and CX3CL1 were elevated in patients with ACS compared with patients with stable coronary artery disease, and the TC levels in ACS patients of the chemokines CCL2, CCL5, and CX3CL1 were reduced by treatment with colchicine. It was also reported that in monocytes isolated from healthy donors, colchicine was able to inhibit the gene expression of CCL2.

By binding to its receptor CCR2, CCL2 induces dimerization and internalization of the receptor–ligand complex, promoting the expression of monocyte chemoattractant protein-1 inducible protein-1. This process triggers the transcription and expression of CCL2, IL-1, and TNF, in addition to activating several intracellular signaling pathways, including JAK/STAT, PI3K/MAPKs, and PI3K/Akt/ERK/NF-κB [46]. These cascades are involved in the regulation of transcription factors and genes associated with cellular processes such as cytokine production, cell survival, migration, apoptosis, inflammation, and angiogenesis [47–50].

Studies have already demonstrated the participation of this axis in the pathophysiology of several CVDs [51,52], making it essential to investigate therapeutic targets based on CCR2-CCL2 signaling. Furthermore, a continuing study by Capers and colleagues [16] revealed that Ang II stimulates CCL2 gene expression. In experimental models of hypertension, Ang II administration resulted in increased expression of this chemokine in the aorta, an effect directly mediated by activation of the Ang II Type-1 receptor [53].

The relevance of the CCR2-CCL2 axis in hypertension has also been demonstrated in animal models deficient for the CCR2 receptor, in which macrophage infiltration into the arterial wall was significantly beneficial, accompanied by a significant decrease in vascular hypertrophy [54]. Similarly, inflammation and vascular remodeling caused by Ang II were attenuated in CCR2^-/-^ mice and in mice undergoing bone marrow transplantation with selective deficiency of CCR2 in leukocytes (BMT-CCR2^-/-^) [54].

Other studies associated increased expression of CCL2 in pulmonary endothelial cells with PAH. CCL2 has been identified as an important mediator in monocyte adhesion and recruitment. In a direct mechanism, CCL2 induces the expression of adhesion molecules, such as E-selectin and ICAM-1 [55] and promotes the expression of endothelin 1 [56], contributing to chronic inflammation, vascular remodeling, and vasoconstriction, characteristic of endothelial dysfunction observed in PAH.

In a clinical setting, a study involving 740 hypertensive patients revealed elevated serum levels of CCL2, which correlated with the severity of organ damage [57]. Furthermore, CCR2 expression on circulating monocytes was significantly increased in hypertensive patients, alleviated after treatment with Ang II receptor blockers [54].

Blocking CCR2 and partially inhibiting CCL2 binding, as a plausible therapeutic target, could prove valuable in managing hypertension. However, the off-target effects of a CCR2 antagonist are poorly understood in the cardiovascular science.

### CCR5

The chemokine CCL5 (also called RANTES) is one of the most important chemokines secreted by macrophages, VSMCs, endothelial cells, and T cells [58–60]. Although it has a higher affinity for the CCR5 receptor, CCL5 can also bind to other receptors, such as CCR1, CCR3, and CCR4 [58,59,61,62]. In CVDs, high levels of CCL5 have been demonstrated, identified in hyperlipidemia [63], atherosclerosis [64,65], and hypertension [7,66].

Blockade of CCL5 or its highest affinity receptor (CCR5) significantly attenuates the atherosclerosis process [67–69], as well as vascular inflammation in cases of lipodystrophy [70] and inflammation of perivascular adipose tissue (PVAT) in hypertension [66], as demonstrated in *in vivo* and *in vitro* studies.

Studies indicate that the CKLF1/CCR5 axis activates NF-κB/NLRP3 signaling, resulting in cell death after cerebral ischemia [71]. In addition, CCL5 is reported to be capable of inducing the proliferation and migration of VSMCs through the activation of NADPH oxidase 1 (Nox1) and blocking CCR5 *in vivo* has been shown to decrease vascular hypertrophy [11], demonstrating its strong involvement in the pathophysiology of CVDs and its potential as a promising therapeutic target.

In our previous study with a mouse model of aldosteronism, an increase in circulating levels of CCL5 and in the expression of CCR5 in endothelial cells was observed. These changes were accompanied by endothelial dysfunction, hypertension, and kidney damage. However, mice genetically deficient in CCR5 (CCR5^-/-^) were protected from these adverse effects, suggesting that CCL5/CCR5-mediated signaling is crucial in mediating aldosterone-induced vascular and renal damage [7]. Furthermore, we also found that CCL5 is produced at different levels across cell types, with the highest baseline expression in T cells, followed by macrophages and then endothelial cells. Upon *in vitro* aldosterone treatment, CCL5 production in T cells and macrophages increased modestly and remained higher than in endothelial cells. However, endothelial cells displayed a more than twofold increase in CCL5 production relative to their baseline levels, indicating a heightened sensitivity to aldosterone stimulation. This suggests that endothelial cells may serve as a primary source of CCL5 in a hypertensive vascular environment. We further demonstrated that mesenteric arteries and aorta incubated with recombinant CCL5 exhibited endothelial dysfunction, suggesting that CCL5 might induce vascular injury by acting directly in the vasculature or by modulating the immune response.

The studies discussed above highlight the potential pathological roles of CCL5 and CCR5 in CVDs and hypertension. However, other findings have questioned the effectiveness of this interaction. For example, Krebs et al*.* [*72*] reported that CCR5-deficient mice were not protected from cardiac and renal injury or hypertension induced by DOCA-salt (50 mg) combined with Ang II infusion (1500 ng/kg/min) [72].

Similarly, Rudemiller et al*.* [*19*] found that CCL5 knockout mice exhibited worsened renal outcomes following Ang II treatment (1000 ng/kg/min), without any significant change in BP. Moreover, Mikolajczyk et al*.* [*66*] demonstrated that neither genetic deletion of CCL5 nor pharmacological blockade using a CCL5 inhibitor (met-RANTES) altered BP in an Ang II-induced hypertension model (490 ng/kg/min), although both approaches improved vascular function, reduced vascular reactive oxygen species, and decreased T-cell infiltration into PVAT. These discrepancies may be attributed to differences in the severity of the hypertension models or experimental variables. For instance, Krebs et al*.* [*72*] used a DOCA-salt model combined with a high dose of Ang II, which may elicit more severe pathology. In contrast, Rudemiller et al*.* [*19*] and Mikolajczyk et al*.* [*32*] employed CCL5-deficient mice rather than targeting CCR5 directly.

**Figure 2:**
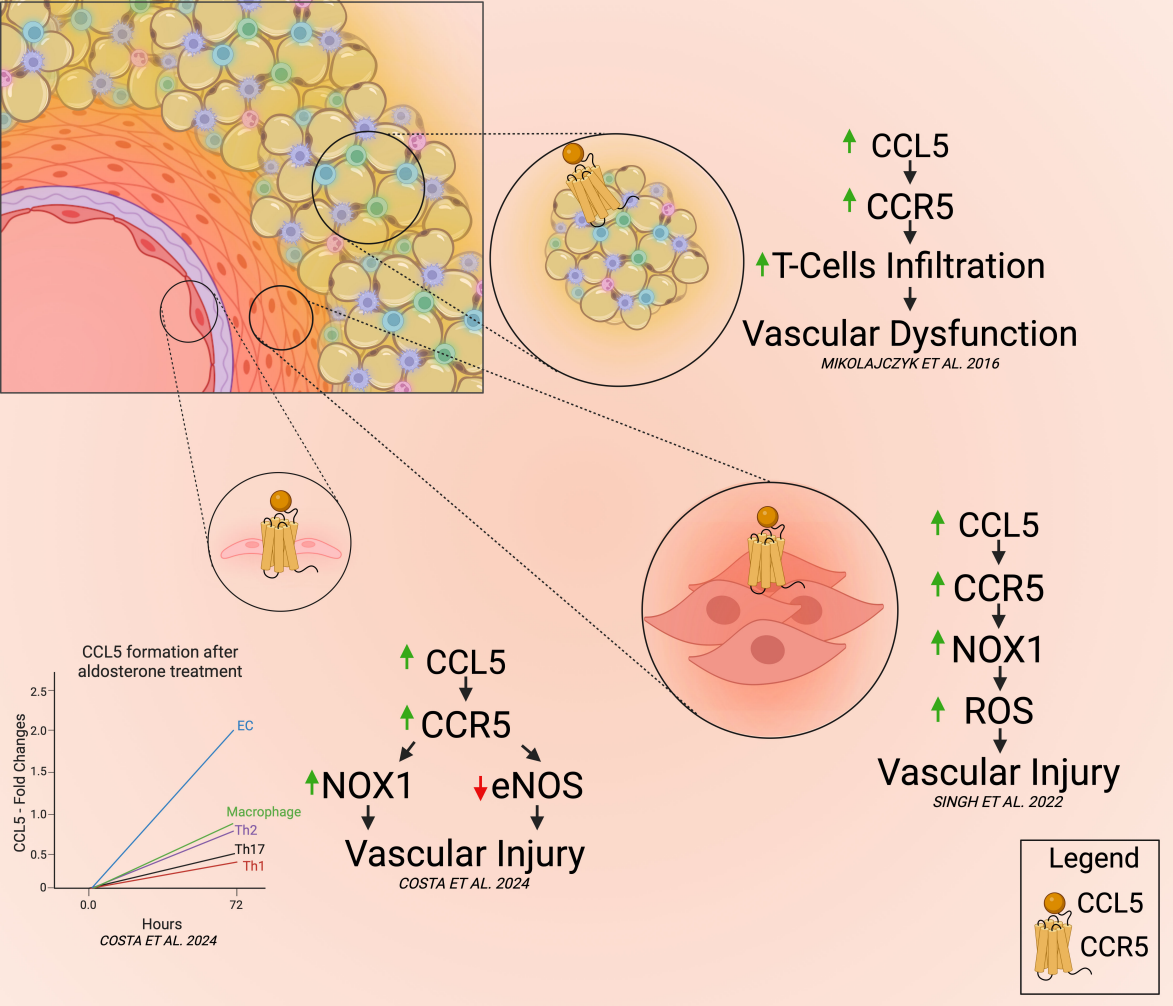
CCL5 signaling through CCR5 contributes to vascular dysfunction, inflammation, and remodeling. CCR5, the primary receptor for CCL5, is expressed in perivascular adipose tissue (PVAT), endothelial cells (ECs), and vascular smooth muscle cells (VSMCs). In PVAT, the CCL5/CCR5 axis promotes T cell infiltration associated with hypertension, contributing to vascular dysfunction. In ECs, CCL5/CCR5 signaling drives inflammation and endothelial dysfunction by dysregulating NADPH oxidase 1 (NOX1) and endothelial nitric oxide synthase (eNOS) activity in the context of hypertension. ECs also appear more sensitive to aldosterone-induced CCL5 production. Additionally, in VSMCs, CCL5/CCR5 signaling activates NOX1, promoting pathological vascular remodeling.

It’s also important to note that CCR5 can be activated by chemokines other than CCL5, such as CCL3 and CCL4, meaning that these studies do not exclude the potential contributions of other CCR5 ligands. Additionally, none of these investigations utilized an aldosteronism model to induce hypertension and organ damage, suggesting that the involvement of CCR5 might be specific to aldosterone-driven hypertension rather than Ang II–mediated models. Further research is needed to clarify these differences across various hypertension models, including the two-kidney, one-clip model and genetic models. Figure 2 outlines the role of this receptor in the process of vascular dysfunction and injury.

It is evident that additional research is needed to clarify the role of CCR5 in hypertension, particularly given the discrepancies observed depending on the mouse model used and the method employed to inhibit CCR5 activation. Moreover, further investigation is essential to validate these findings in clinical settings, such as by analyzing circulating levels of CCR5 ligands. Finally, due to CCR5’s lack of specificity for CCL5, identifying the key ligands responsible for CCR5 activation in CVDs and hypertension would be highly valuable for advancing the literature.

### CXCR1 and CXCR2

Like the other receptors already discussed, CXCR1 and CXCR2 play a crucial role in the development of several CVDs, such as atherosclerosis, ischemia, cardiac remodeling, and hypertension. CXCR 1 ligands include CXCL6 and CXCL8 at high concentrations as well as CXCL1, CXCL2, CXCL3, CXCL5, and CXCL7, while CXCR2 ligands include migration inhibitory factor (MIF), CXCL1, CXCL2, CXCL3, CXCL5, CXCL6, CXCL7, and CXCL8 [73]. In this section, we will discuss the main pathophysiological characteristics associated with these receptors and the development and progression of CVDs. Both receptors have a high level of homology between them, which is why most studies investigate both receptors together.

The CXCR2 receptor plays a crucial role in the development of atherosclerosis through the chemotaxis it exerts on the cells of the immune system, since the condition of hypercholesterolemia causes neutrophilia, and in this case, CXCR2-dependent neutrophilic infiltration is transmitted in aortic lesions [74].

In LDLR^-/-^ (LDL receptor knockout) animals, CXCR2 deficiency in the bone marrow reduced the severity of atherosclerotic lesions [75]. Furthermore, unconventional ligands can intensify the inflammatory activity of CXCR2 in the arterial wall. Macrophage MIF, for example, acts as an agonist of this receptor, facilitating the recruitment of monocytes to atherosclerotic plaques [76].

CXCR2 also influences monocyte homeostasis in response to high-fat diets, affecting disease progression [77]. Another mechanism involves the activation of perivascular mast cells, which promote the adhesion of monocytes to atherosclerotic plaques through the interaction between CXCR2 and VCAM-1, aggravating the progression and destabilization of lesions [78]. Furthermore, the activation of CXCR2 by dioxin derivatives can induce a vascular inflammatory response, mediated by the aryl hydrocarbon receptor, reinforcing its participation in atherogenesis [79].

On the other hand, CXCR2 is also involved in vascular healing. Its activation can contribute to endothelial recovery after injuries, including those caused by atherosclerosis [80–82]. This suggests that, although CXCR2 has a clear atherogenic role, it can also influence vascular repair processes.

Another important point is that CXCR2 also plays an important role in hypertension, vascular dysfunction, and cardiac remodeling associated with the disease. The levels of this receptor are elevated in the hearts of mice infused with Ang II [83] as it was also seen in spontaneously hypertensive rats (SHR) [84], and when using the AT receptor antagonist, candesartan, a decrease in protein of this receptor was seen, which suggests a connection between it and the RA system.

In addition, in SHR animals, the BP increasing in this model has a certain link with the infiltration of macrophages in the cardiovascular system [84], and the deletion of the CXCR2 gene in mice attenuates hypertension, reduces monocyte infiltration in the heart, and improves cardiac remodeling [83,85]. Furthermore, decreased transcription factor Krüppel-like factor 15 (KLF15) in Ang II-infused mice is associated with increased expression of CXCL1 and CXCR2, aggravating cardiac dysfunction and remodeling [86].

In a comprehensive and impactful study, Wang et al. [85] demonstrated that CXCR2-deficient mice are protected from Ang II–induced vascular dysfunction, remodeling, inflammation, and hypertension. The role of CXCR2 was further validated pharmacologically, as treatment with a CXCR2 antagonist similarly mitigated these effects in Ang II–treated mice*.* Notably, CXCR2’s contribution to hypertension appears to originate from the immune compartment transplantation of CXCR2-deficient bone marrow into wildtype mice conferred protection against Ang II–induced vascular injury and hypertension, whereas transplantation of wildtype bone marrow into CXCR2-deficient mice did not. In humans, the relevance of these findings is supported by elevated levels of CXCR2^+^ pro-inflammatory cells in hypertensive patients compared to normotensive individuals [85] and by the association of these cells with atrial fibrillation [62].

Nowicki et al. [87] demonstrated that CXCR1/2 blockade may present a promising therapeutic strategy to prevent the progression of cerebral aneurysms. In their study, he demonstrates that signaling mediated by the platelet-driven CXCL7-CXCR1/2 inflammatory axis plays an essential role in the formation and rupture of cerebral aneurysms in mice. Inhibition of this axis reduces neutrophil infiltration into the vascular wall, attenuates inflammation, and prevents both the development and rupture of aneurysms.

The CXCR2 receptor is notably up-regulated in hypertensive conditions, particularly in models using Ang-II agonists, and appears to play a pro-atherogenic role in the cardiovascular system. While CXCR2 antagonists show promise in reducing hypertension, their beneficial effects likely arise from actions on immune cells. Therefore, developing strategies that selectively target specific immune cell populations could offer significant advances in the cross-talk between immune activation and hypertension.

### CXCR4

CXCR4, another chemokine receptor, plays a crucial role in the infiltration of inflammatory cells into the heart after myocardial infarction, significantly contributing to the inflammatory process and subsequent cardiac remodeling [88,89]. Furthermore, more recent studies indicate that CXCR4 is also involved in the diastolic cardiac dysfunction observed in heart failure with preserved ejection fraction [90]. In this context, CXCR4-mediated signaling between macrophages and fibroblasts is a key mechanism for disease progression.

The SDF-1/CXCR4/CXCR7 pathway has attracted increasing attention in cardiovascular research, being associated with several cardiac pathologies. Indeed, activation of the SDF-1/CXCR4 axis has been shown to contribute to inflammation in both the heart and kidney in DOCA-salt-induced hypertension models, while inhibition of CXCR4 with AMD3465 resulted in improvement of myocardial fibrosis, renal fibrosis, and left ventricular remodeling, besides reduction of BP when DOCA-salt rats were treated with AMD3465 [91].

Additionally, studies demonstrate that dysregulation of this signaling is involved in pregnancy-related diseases, such as preeclampsia, suggesting a key role for CXCR4 also in gestational hypertensive disorders [48,49]. The elevated expression of CXCR4 in chorionic villous tissue during the first trimester of pregnancy is indicative of its relevance in the development of hypertensive complications in pregnancy [48,49].

Thus, the CXCR4 pathway is not only closely linked to the pathogenesis of several CVDs, but due to its role in the pro-inflammatory signaling response, it may be a promising therapeutic target for conditions such as heart failure, hypertension, and pregnancy-associated complications.

**Figure 3:**
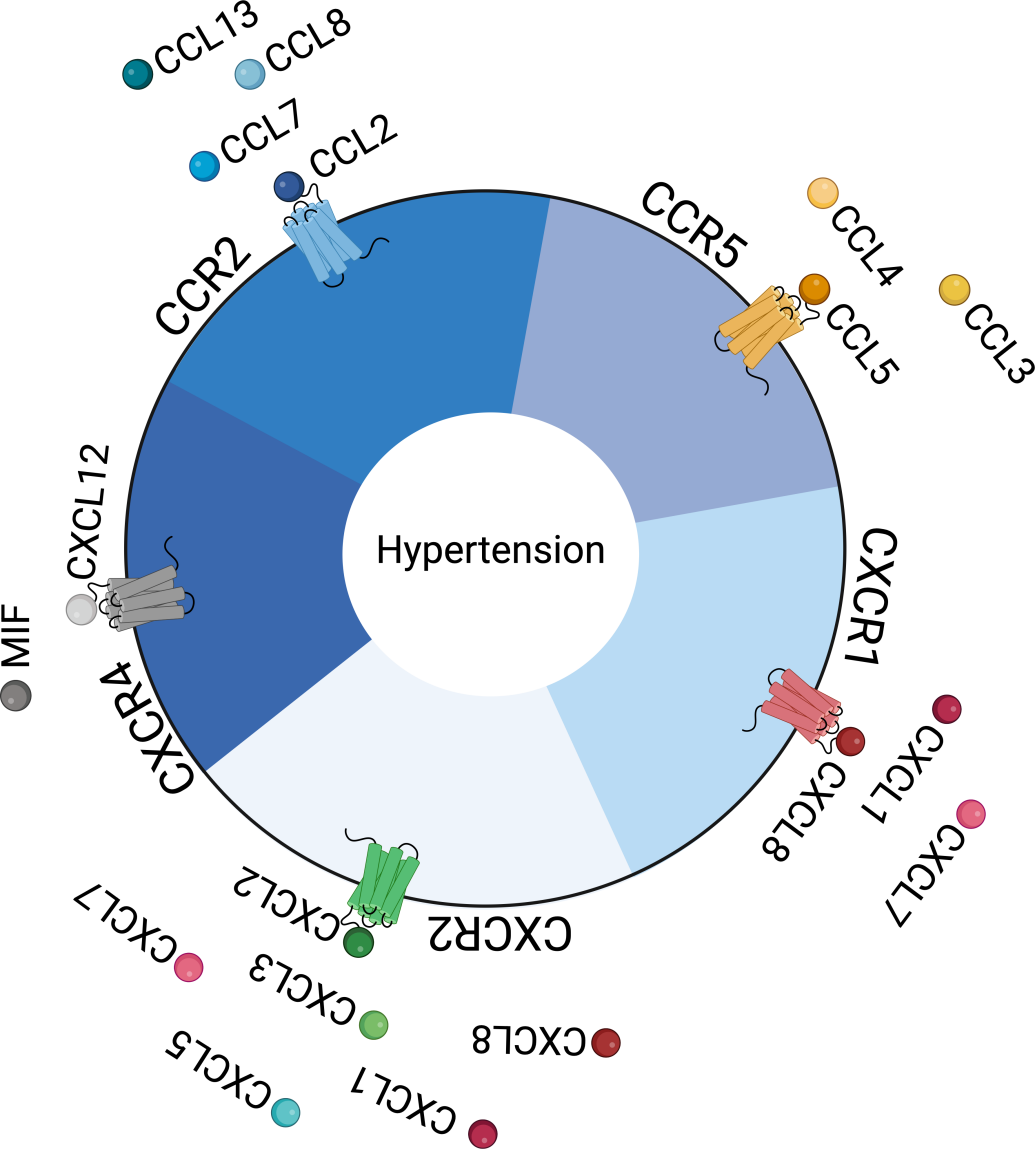
Key chemokines and their receptors in hypertension. In hypertension, various chemokine receptors and their ligands are up-regulated across different organs and cell types. This figure summarizes current knowledge regarding chemokine–receptor interactions. Receptors are positioned along the edge of the central circle, while their corresponding ligands (represented as circles) are located outside the circle. Color coding indicates the specificity and proximity between each ligand and its receptor.

In Figure 3, we schematically illustrate the main receptors mentioned in this review article and their activators, summarizing the information discussed in this section.

## Pharmacological approaches

Given the central role of chemokines and their receptors in the pathophysiology of hypertension, these components have been consolidated as promising therapeutic targets for the control and progression of the disease. Several compounds have been developed or even repositioned for off-label use, acting on the modulation of the receptors discussed in the previous sections, with the aim of reducing inflammation and/or restoring tissue homeostasis.

In the following section, we summarize key pharmacological agents, their molecular targets, and potential therapeutic implications in the context of hypertension (Table 2). This overview provides a foundation for understanding how manipulation of chemokine signaling may contribute to future clinical interventions.

**Table 2:** Therapeutic targets and respective information on selectivity, cardiovascular effects, and adverse effects

Molecule	Target	Mechanism	Adverse effects	References
RS504393	CCR2	Selective CCR2 receptor antagonist, inducing improvement in renal function, preventing increased inflammation and reducing BP in hypertensive models induced by DOCA-salt.	No adverse effects studied.	[92–94]
INCB3344		Selective CCR2 receptor antagonist; demonstrated protective effect against hypertension in DOCA-salt animals, reducing CCR2 mRNA expression, reduces influx of M2 macrophages into the vessel wall and prevents arterial fibrosis.	No adverse effects studied.	[95,96]
Maraviroc	CCR5	Selective antagonist of the CCR5 receptor, it is capable of reducing renal fibrosis and infiltration of CD4 T cells. In endothelial cells, it was able to reduce reactive oxygen species, decrease NFkB activation, reduce the expression of NOX1 (NADPH oxidase 1 complex), and restore nitric oxide production.	The most commonly observed side effects include: diarrhea, nausea, headache, dizziness, cough, insomnia, skin rashes, weakness, muscle aches, abdominal pain.	[7,97,98]
Reparixin	CXCR1 CXCR2	Non-selective inhibitor of CCR1 and CCR2, capable of reducing BP, increasing blood flow, and reducing arterial wall thickness in SHR animals; In C57BL/6 mice, it was able to reduce the formation of cerebral aneurysms.	The most common adverse effects include mild to moderate gastrointestinal symptoms and fatigue.	[87,99,100]
SB225002	CXCR2	Selective CXCR2 antagonist. Its use is capable of reducing BP, reducing the duration of arterial fibrillation and promoting atrial remodeling, in addition to decreasing macrophage recruitment, reducing superoxide production associated with multiple pathways, such as TGF-b1/Smad2/3, NFkB-p65, NOX1, NOX2, Kir 2.1, Kvl.5, and Cx43, in SHR rats. It has also been reported to reduce fibrosis and pulmonary hypertension in mice.	No adverse effects studied.	[60,62,84,101,102]
SB265610		Selective CXCR2 antagonist capable of reversing dysfunction, cardiac remodeling, and inflammation induced by Ang II infusion in KLF15 knockout mice.	No adverse effects studied.	[86]
AMD3100 (Plerixafor)	CXCR4	Selective CXCR4 antagonist, promoting inhibition of the SDF-1/CXCR4 axis, which significantly improved lung alveolarization and decreased pulmonary hypertension, right ventricular hypertrophy, vascular remodeling, vascular cell proliferation, and lung or right ventricular stem cell expressions in murine model.	It presents mild and transient symptoms such as gastrointestinal symptoms, headache, dry mouth, and paresthesia.	[103,104]
AMD3465		Selective CXCR4 antagonist, in DOCA mice, coadministration of a CXCR4 antagonist, AMD3465, caused a marked reduction in BP	No adverse effects studied.	[91]

Although many of the reviewed compounds demonstrated promising results in preclinical models, it is important to point out several limitations that must be considered before translating these findings into clinical practice. First, most chemokine receptor antagonists are non-target specific, meaning off-target effects can impair other physiological functions, particularly those mediated by chemokines. This limited selectivity can affect treatment efficacy and complicate safety considerations, particularly due to the redundancy and overlap among chemokine receptors and their ligands [26,33]. As a result, potential side effects may go unrecognized due to the limited clinical evaluation of many chemokine receptor antagonists. For instance, RS504393 and INCB3344 lack published safety data beyond studies in rodent models [92–96]. In contrast, maraviroc [97] has a well-characterized safety profile, with reported adverse effects including gastrointestinal disturbances, dizziness, and hepatic toxicity. The absence of long-term toxicity assessments and the unknown risk of immunosuppression further raise concerns. Chemokine signaling is essential for immune surveillance, host defense, and tissue repair. Thus, broad inhibition of chemokine receptors may compromise immune function, increasing susceptibility to infections or impairing wound healing—as seen in models of CCR1 or CCR2 antagonism during viral infection [19,66].

In addition to systemic risks, context-dependent effects of chemokine receptor antagonism are evident in various hypertension models. Some receptors, such as CCR5 or CXCR2, exhibit dual functions, participating in both tissue injury and repair, depending on the disease stage and cell type involved [66,72,85], which highlights the importance of developing precision-targeted therapies, potentially using delivery systems that restrict drug activity to specific cell types or tissues and minimize systemic immunosuppression.

As mentioned earlier, while the main pharmacological focus has been on compounds directly related to arterial hypertension, other agents with effects on relevant receptors are under investigation in preclinical models. For instance, TAK-779, an antagonist of the CCR5/CXCR3 receptors, has shown anti-inflammatory activity in models of inflammatory, infectious, and autoimmune diseases [105], including HIV [106], renal injury [107], and diffuse lung damage [108]. A study demonstrated that TAK-779 reduced macrophage infiltration and inflammatory cytokine production in models of renal ischemia-reperfusion injury [96], suggesting potential for modulating renal inflammation. Although there is no direct evidence linking TAK-779 to BP control, its immunomodulatory effects may indirectly influence hypertensive mechanisms, particularly those involving inflammation. These findings support further investigation into novel molecules targeting chemokine pathways for more effective BP regulation.

While chemokine receptor antagonists hold promise for treating CVDs, their use requires caution due to the critical role chemokines play in immune regulation. Chemokines are essential for directing immune cell migration and activation, particularly during infections. Preclinical studies have shown that disrupting these pathways can compromise host defense. For instance, mice lacking CCR1, CCR2, or CCR5 exhibited severe disease and increased mortality following MA15-SARS-CoV infection, due to impaired immune cell recruitment to the lungs. Similar findings were observed in human dendritic cells infected with SARS-CoV [109]. Additionally, blockade of CCR1 and CCR5 in models of Herpes Simplex Virus Type 2 infection impaired host defense and altered the cytokine response, indicating a protective role for these receptors during infection [110]. These examples emphasize the need for careful evaluation of chemokine-targeted therapies in clinical settings, as unintended immunosuppressive effects could pose serious risks. Selective targeting approaches and immune monitoring will be critical to ensure these therapies are both effective and safe for hypertensive patients.

## Therapeutic strategies under development

### Monoclonal antibodies against chemokines/receptors

The use of monoclonal antibodies to block chemokines and their receptors has been investigated as a promising therapeutic approach for the treatment of hypertension. Anti-CCL2 (Carlumab), for example, has shown potential in reducing inflammation in several conditions [111–113], although it has not been extensively tested in models of hypertension. However, as previously mentioned, the CCL2/CCR2 axis has been well characterized in the pathophysiology of hypertension, especially for its involvement in the infiltration of monocytes/macrophages into blood vessels [45,51].

Other monoclonal antibodies are also undergoing clinical trials acting on the CCR2/CCL2 pathway. For example, MLN1202 is a highly specific humanized monoclonal antibody that interacts with CCR2 and inhibits CCL2 binding. MLN1202 treatment was well tolerated in this patient population at risk for atherosclerotic CVD and resulted in significant reductions in high-sensitivity C-reactive protein levels [114]. However, it did not find significant results in inflammatory diseases, as shown in the study on rheumatoid arthritis patients by Vergunst and collaborators [115]. Despite the interesting findings, the limited sample size compromises the statistical robustness of the results, making broader generalizations difficult. As emphasized above, the clinical use of immune response modulators in CVDs must be carefully evaluated to ensure both efficacy and safety.

## Conclusion

In summary, as emphasized throughout this review, chemokines and their receptors represent promising therapeutic targets for CVDs, particularly hypertension. These molecules contribute to cardiovascular damage through a dual mechanism: directly inducing vascular injury and promoting the recruitment of immune cells that exacerbate tissue damage. Consequently, targeting chemokine signaling pathways holds potential for reducing inflammation and mitigating vascular injury—key elements in the progression of these conditions. Although preclinical studies have shown substantial progress, translating these findings into clinical practice presents several challenges, including achieving receptor specificity, ensuring drug selectivity, and avoiding unintended suppression of essential immune functions; for instance, dampening the immune response could significantly impair the body’s ability to fight off something as common as a cold.

Given the complexity of immune responses and their implications for other pathologies, more rigorous preclinical and clinical studies are essential to validate the safety and efficacy of chemokine-targeted therapies. Future investigations should also consider the interactions between these interventions and coexisting conditions in order to develop more precise, effective, and safer treatments for hypertension and other CVDs.

## Abbreviations

Ang II: angiotensin II
BP: blood pressure
CVD: Cardiovascular disease
RAAS: renin–angiotensin–aldosterone system
VSMC: vascular smooth muscle cell
